# Comparing the Art of Midwifery Practice to Spiritual Care: Common Characteristics Emerging from Theoretical Models and Field Work

**DOI:** 10.1177/15423050261483272

**Published:** 2026-08-31

**Authors:** M-N Bélanger-Lévesque, S Crowther

**Affiliations:** 1 1410 Faculty of Health and Environmental Sciences, Auckland University of Technology, Auckland, New Zealand; 2 7321 Centre d'étude du religieux contemporain, Université de Sherbrooke, Sherbrooke, Canada

**Keywords:** Spiritual care, midwifery, theoretical models comparison

## Abstract

This article offers an original comparative analysis of spiritual care providers and midwives using two theoretical models, the “art of midwifery practice” and “practiced spirituality”. The analysis draws on interview data from ten New Zealand midwives. Focusing on presence, guardianship, intuition, confidence/courage, and human relationship. The analysis reveals shared yet distinct ways of enacting care, illuminating overlooked dimensions of spiritual care practice, and opening new avenues for interdisciplinary reflection.

## Introduction

Spiritual care providers in healthcare institutions are regularly asked in clinical and academic settings to position themselves in relation to other helping professionals. This identification of professional specificity through a process of comparison is inevitable and helpful; nonetheless, the structuring influence of specific professions regarding spiritual care raises concerns because the scope of the comparison is frequently limited to the psychosocial field. For example, a guide to oncology and palliative care clarifies the distinct role played by spiritual care providers, psychologists, psychiatrists, sexologists, and social workers. (Ministère de la Santé et des Services sociaux, 2017)

The case of Quebec, the francophone Canadian province where the first author resides, provides an interesting insight. If spiritual care was previously provided in healthcare establishments by chaplains through agreements with the main religious authorities (mostly Catholic), a 2011 unilateral decision of the Ministry of Health transferred this under public responsibility, with the creation of the professional title of “spiritual care providers”. This deconfessionalization and secularization entailed a process of professionalization of the practice, raising questions of professional identity as those involved in spiritual accompaniment seek their specificity from other forms of professional support. Critics have questioned the “psychologization” of the profession, the displacement not only of language (“quest for meaning”, “spiritual needs”, “spiritual distress”) but also of practices (rituals, teaching) away from religion, adding that the influence of a certain technoscientific logic and bureaucracy of health based on productivity and efficiency could have harmful impacts on practice with a loss of their professional specificity. (Cherblanc & Jobin, 2013, 2020)

To better define their professional status, spiritual care providers, specifically those involved in hospital and palliative care chaplaincy, can gain a lot by turning to a profession outside the psychological field: in this case midwifery. An obvious reason to support the relevancy of such comparison is the historical presence of midwifery and of spiritual care, having been around for much longer than any institutional structures. Childbirth and spiritual care providers across cultures have always been there to offer support to others through life transitions, notably through the performance of rituals (Butler et al., 2025; Cheyney & Davis-Floyd, 2021; Hess, 2024). However, like spiritual care, midwifery still faces contemporary challenges in its encounter within the medical domain: “Midwives’ increased autonomy and overlap into the medical domain has perhaps encouraged the use of the dominant medical discourse to form and define practice.” (MacLellan, 2011, p. 26) More existentially, both practices are constantly dealing with questions on suffering and death but also living spiritual experiences of joy and awe. (Wojtkowiak & Crowther, 2018)

Literature on spiritual care inside midwifery is gaining momentum (e.g., Crowther et al., 2021; Crowther & Hall, 2017; Hall, 2016; Karabey et al., 2025; Lemay & Hastie, 2018; Linhares, 2012; Ross & McSherry, 2025), however, comparisons between the practice of spiritual care providers and midwives have been largely neglected. Our literature review revealed no field study on the topic and few reflective texts in pastoral care. Crowther (2019) identified the need to create multidisciplinary ways of working in maternity that includes hospital chaplaincy and midwifery and opening a dialogue that would help cultivate an art of spiritual care around childbirth. Knowing our differences as well as where these two professions converge is worth consideration. In *Minister as midwife,* Hanson (1996) explores the notion of midwives and hospital chaplains being parallel professions, drawing on scripture, history, and personal experience to support the idea, whereas Haig present “the image of god as one who midwifes us into new beginnings suggests a relational, co-constructing activity of a different quality from that of the theistic Father or Creator God”. (2010, p. 1)

The aim of this paper is to explore common, yet distinctive, characteristics of spiritual care, in relation to midwifery by comparing two theoretical models and by offering illustrations of those qualities from a research study with midwives.

## Methods

### Comparison of Theoretical Models

Our first approach compared theoretical descriptions of these professions. More specifically, the “art of midwifery practice” as described by MacLellan (2011) was selected as a theoretical model created from a discourse analysis of literature review surrounding skills attributed to midwives and women's quality of care. MacLellan goal is to render visible the intangible skills used by midwives which “reflect the art of midwifery [that] often goes unnoticed but carries huge value among its recipients and embodies the philosophy of the profession”. (MacLellan, 2011, p. 26) Four concepts of fundamental skills were identified; presence, guardianship, intuition, and confidence and courage, with a fifth, “human relationship” as the glue that binds this “art” of midwifery or the weave of the tapestry that holds maternity care together (Hunter et al., 2008).

These characteristics were compared to a recent grounded theory around the “*spirituel pratiqué*” (practiced spirituality) of spiritual care providers in Quebec, known elsewhere as hospital chaplains, working in either long-term care facilities or hospitals, and often but not solely in palliative context. This research is based on a qualitative data collection of professional life stories (*récits de vie*), an approach inspired by ethnomethodology (Cherblanc & Jobin, 2020). What was described is first and foremost a functional, practice-based spirituality, as distinguished from a substantial representation of spirituality; it is a pragmatic representation of spirituality, interested in its effects rather than in its origins. It fits with a universalist representation of spirituality, which posits that spirituality is a phenomenon that is not linked to the boundaries between different traditions; that spirituality is universal and that traditions (religious and spiritual) are historical phenomena. As such spirituality is acknowledged as a living-experience of being human that may or may not align or be informed by an established and/or named religion or spiritual tradition.

#### Study Contextual Differences

In midwifery and spiritual care, Quebec and New Zealand offer contrasting yet rich contexts shaped by their histories, healthcare systems, and cultural values. Quebec, a francophone province within bilingual Canada, operates under a publicly funded, secular healthcare system influenced by French civil law and strong cultural protectionism. Midwifery in Quebec emphasizes clinical autonomy and evidence-based practice, with spiritual care generally treated as a personal or optional aspect, reflecting the province's commitment to laïcité (secularism) (Gagnon & Lemay, 2025). In contrast, New Zealand's midwifery model is grounded in partnership and continuity of care, deeply informed by its bicultural foundation and the Treaty of Waitangi (Stevenson et al., 2020). The healthcare system is also publicly funded but integrates Māori perspectives, where birth is considered a sacred event and wairua (spiritual wellbeing) is central. Spiritual care providers often collaborate with midwives, especially in Māori and Pasifika communities, offering holistic support that includes emotional, cultural, and spiritual dimensions (HRCNZ, 2023). While both regions share British-influenced healthcare structures due to their Commonwealth ties, New Zealand's integration of indigenous spirituality into maternity care sets it apart from Quebec's more secular and culturally distinct approach, highlighting how colonial legacies and indigenous relationships shape care in profoundly different ways (Barnabe, 2021).

#### A Study on “Presence as Spiritual Care” Around Childbirth

To deepen this comparison, we drew from the first author's postdoctoral research on “presence as spiritual care” around childbirth conducted in Aotearoa/New Zealand where more than 80% of pregnancies and births are attended and supervised by midwives as the primary professional carer (Grigg & Tracy, 2013). The primary research complete analysis (forthcoming) is informed by hermeneutical phenomenology where lived-experience (*Erlebnis*) is the starting point of inquiry into the human existence (*Dasein*), where data is interpreted to surface meaning which facilitates the sharing of human experiences in ways that resonate with listeners/readers (Van Manen, 2016). This is achieved through a series of descriptive and interpretive processes that culminate in revealing an emergent phenomenon (Crowther et al., 2017). In thisdiscussion article, the gathered data is presented in a more exploratory and descriptive way, to inform the professional comparison.

Following ethical approval from Auckland University of Technology Ethics Committee (23/30), participants were purposefully selected through word of mouth, professional email groups, social media, and snow-ball technique, with an aim of maximum variation to ensure a diversity of voices (Dibley et al., 2020). All 10 participants were practicing Aotearoa/New Zealand midwives at the time of data collection. Data collection involved dialectical open interviews at a chosen location and time depending on the place they might be more comfortable. Interviews were individual except for one dyadic; two were virtual and others in person at various locations throughout the country and varied in length between 40 to 90 min. For this article we focus on specific findings from interview verbatim transcripts that were crafted into stories at the first level of descriptive analysis (Crowther et al., 2017); the stories selected for their deepening and illustration of each theoretical characteristic of midwifery foreground the spiritual care given by midwives, in their own words.

### Reflexivity

Congruent with the methodology, acknowledgement of the authors’ position toward the topic is crucial as our preconceived understandings contribute to the conditions of emerging interpretation; there is hence a need to be explicit to provide evidence of trustworthiness of the method. (Blix, 2011; Koch, 1996; Powell Kennedy et al., 2010; Smythe et al., 2008) Bélanger-Lévesque, PhD entered religious studies research with a passion to deepen her understanding of her four childbirths; her choice for this postdoctoral subject is supported by a desire to better equip healthcare professionals to consider spirituality around childbirth. Crowther, PhD, a leading figure in research on spirituality and childbirth with a solid background in hermeneutic phenomenology, accepted to act as a supervisor. Both authors leaned into their clinical practice experiences to substantiate and inform the data analysis: Bélanger-Lévesque as a spiritual care provider in the public healthcare system in Quebec for the last 5 years, and Crowther's experience of more than 30 years as a midwife working across practice areas, leadership roles, academia, and research.

## Findings

Each of the characteristics of MacLellan's art of midwifery practice are reprised here separately with i) a short description of its attributes; ii) illustrations with crafted stories extracted from interview transcripts with midwives; and iii) a comparison to the spiritual practice given by Cherblanc and Jobin (2020) study participants. Findings are summarized in Table 1.

**Table 1.** Compared Characteristics of the art of Midwifery Practice Model (MacLellan, 2011) and of the practiced spirituality in spiritual care (Cherblanc & Jobin, 2020).

Compared Characteristics	Attributes in the Art of Midwifery Practice (MacLellan, 2011)	Similarity in the Practiced Spirituality in Spiritual Care (Cherblanc & Jobin, 2020)
Presence	Therapeutic social relationship, reassurance, guide Facilitating the women's confidence and her process Watchful waiting	Support (accompaniment) Comportment and attitude supporting one's empowerment Taking the time, non-action Non-directive listening, silence
Guardianship	Security, safe-keeper, resisting urge to control, trustworthiness, familiar Physical skills and advice	Trustworthiness in a relation of equals Creating a space for one's spirituality, ritual Ethical and moral advice
Intuition	Subconscious knowing, insight, cluefulness, reflection embodied knowledge Emotional connectedness, partnership, mutuality	Intuitive intervention, difficult to describe Non-directive accompaniment Physical touch
Courage and Confidence	Courage and confidence to make decision, accept accountability Belief the women can do it, positivity, praise, encouragement Acceptance of uncertainty/uniqueness	Empowerment Positive change in the person Recognition expressed by patients, families and healthcare professionals.
Human Relationship	Compassion, empathy, caring, gentleness, communication, patience, quietness, calm Hands-on high touch technique	Bond of trust, empowerment, guide Mobilizing one's spiritual resources Facing one's suffering and losses, illness and death peacefully

### Presence

Presence is the first characteristic of the art of midwifery practice described in MacLellan's theoretical model and has three attributes. First, presence is much more than being physically in the room, it is an “engaged presence”, it is becoming a reassuring and comforting guide. Second, it is the midwife's belief in the natural process of childbirth, that the midwife facilitates the woman's confidence and ability to birth her baby. Third, presence is also “the art of doing nothing well” in watchful waiting, in this confidence not to act and to become a care tool in herself.

A story of presence:

I was in a caesarean once where I’m not doing anything – I’m just observing. The baby was taking a long time to be delivered – I could see everybody start looking at the clock, I know that they’re getting stressed preparing the conversations are spoken, and non-spoken, I can see and feel the anxiety lifting in the room. In that situation, what can I do? It sounds strange to say it, but you can change the atmosphere of a place, just by your intentions, by what you bring. I just stood there physically, like a power stance, and just change it, just by taking deep breaths, and saying to myself: “Okay, with every breath, I’m changing this – we’re not all just going to get hyped-up – we’re just going to calm it down.” Whether I’m the only one experiencing that or other people are experiencing that, I don’t know, I also don’t mind; if I’m in my own space in my own delusion, at least I’m not getting on the crazy train. A big part of spiritual care that I love is this way of being there. Eva.

Likewise, for the spiritual care providers of Cherblanc and Jobin's study, presence is described in terms of support (*accompagnement*): the specificity of the practice is not so much the intervention tools but rather the comportment and attitude in relation to the person's empowerment. It takes time for presence between equals and for sympathetic listening to unfold. The aim of being present is to let the person take the direction of the intervention and realize what strengths and capacities reside inside. The particularity of listening in spiritual care is its non-directive and non-solution-driven approach like other professionals in the healthcare system. Being present takes precedence over a vision of the helping relationship focused on words: the distinctive practice of spiritual care providers is around time, listening and silence, and for some, physical contact to signify presence and proximity.

Presence allows and invites support marked by the non-verbal and by *non-action*. In clinical practice, this is especially true when spiritual care cannot be addressed through the quest for meaning/purpose, often identified as a major role of the profession. For example, with a person with significant cognitive losses the spiritual care provider is “just there”: it is a question of resisting the urge to intervene to give time to another, this time is necessary to create trust. Like the midwife, the healthcare spiritual care worker is the care tool: spirituality as such invites both the care givers and the care receivers to be watchful and receptive of presence (to oneself, to others/Other).

### Guardianship

In MacLallan's model, the notion of “guardianship” in midwifery is the product of a relationship of mutual trust; and constitutive of two attributes. First, being a guardian is creating a safe space, to protect the natural process of birth, nurturing a sense of security and protection both for the delivering woman through trustworthiness and to the midwife by resisting the urge to want to control childbirth. Second, guardianship rests on the woman's confidence in the midwife's ability to intervene if necessary, ensuring that the process is not continually disrupted by comings and goings and other disturbances. Nonetheless, if the midwife has the skills and knowledge, the emphasis is placed on the empowerment of the woman who gives birth, in her choices, empowerment, satisfaction and self-realization.

A story of guardianship:

The life of a midwife comes with stress and emergency and challenges, but you’re still being able to maintain that mother's safe-space of nurturing that childbirth journey. That lady zoned out in her own way, like an outer body experience. She displayed these actions of being so in-tuned in her body that as a midwife, I could just follow her clues. I needed to do very little but keep a safe space around that woman to ensure that this was her birth, in the way that she wanted it. It's so beautiful to witness a woman trusting and having faith in her own body, and nature, and how things happen. It was very humbling and honouring to be a part of, it was very uplifting and inspiring, and for want of a better word; spiritual. There was a spirit of holding that space, as a midwife for women, and protecting that space. Cathie

The concept of guardianship is not used by spiritual care providers in Cherblanc and Jobin's study. Nonetheless, creating a space for spirituality and/or for the person is stated twice in the paper. If women trust midwives’ technical knowledge, there is a trust toward healthcare spiritual care providers’ abilities, for example maneuvering complex ethical questions or carrying out rituals. Guardianship is particularly visible in the latter, notably when “space” is created when performing end-of-life rituals, a distinctive element of their practice, supporting a different natural process by also reducing suffering.

Like the midwife, and as pointed out by Cherblanc and Jobin, it is more a question of comportment and attitude adopted: the idea of trust builds on a relationship of equals is central to spiritual care providers, as support requires an egalitarian position, therefore not arriving as “one who knows”, but as “one who knows nothing” is central. Thus, spiritual care providers insist on this non-interventionist aspect: there is no “hidden agenda”, and they resist any institutional mandate in order to be there for the person, to know and recognize their thoughts, their representations of suffering, illness or death, its strengths too, its wishes and desires regarding possible and offered care, etc.

### Intuition

Intuition is the third characteristic of the art of midwifery practice with two attributes. First, MacLellan (2011) brings together expressions of subconscious knowledge and embodied knowledge where clues, signs and other types of information are taken into consideration. Experience fuels reflections and reflexivity, clarifying this intuition over the years: this is where art and science merge. Second, intuition builds on the emotional connectivity between caregiver/care recipient, in a partnership and mutuality, accepting uncertainty without losing sight of complications.

A story of intuition:

This lady almost felt silly for calling me: “I just feel a little bit light-headed.” I would never normally, medically speaking, take that person in but I had this strong gut feeling to take her in to check her out. She was perfectly normal: her blood pressure was normal, no headache, she’d been feeling good movement, the baby was well-grown, and the urine sample came back normal. As she stood up to leave, she literally had a placental abruption, and started bleeding all over the floor; luckily, I rushed her down for a C-section and the baby made it fine. God must have put something in her spirit to call me, and then He put something in my spirit to take her in. Hannah

Intuition is only cited once in Cherblanc and Jobin's paper on spiritual care providers, in the form of a verbatim: “My work intervention, it's very intuitive. I couldn’t describe the process to you exactly, nor exactly how it develops in every situation” (*in French in the original)*. They point to elements necessary to intuition: spiritual care providers aim to accompany without directing, to put the person at the center of their intervention, placing great emphasis on the very varied and diversified aspect of their spiritual interventions, all in the goal of empowering. The dimension of physical touch, a distinctive characteristic noted by Cherblanc and Jobin (2020) also relates to intuition, relating to the embodied knowledge described by MacLellan (2011).

It is a topic rarely discussed by the first author with colleagues and in professional circles, yet critical to the practice in healthcare context. A telling example is when one is called urgently for an end-of-life ritual, there is only few minutes to create something significant in the life story of the dying person but also for their loved ones: what appears as a seemingly spontaneous ritual is an intuitive assembling of details given about the person, but more often of unspoken but felt information and dynamics in the room, crafted into a ritual with some selected texts. Intuition tells how to introduce oneself in a room, what is the vibe, etc. It is reasonable to believe that for many spiritual care providers, this ‘skill’ of intuition originates in their own spirituality: a gift or message from God, etc.

### Confidence and Courage

MacLellan brings together both confidence and courage under three attributes. First, courage and confidence are necessary in a context of autonomy where the midwife must both make decisions regarding care, not out of fear, but as to hold herself accountable for her actions. Second, it is also courage and confidence emerging from a midwife's own empowering that facilitates these qualities in the laboring woman to achieve this, which leads her to encourage and praise her. Third, confidence and courage are visible when the midwife accepts the uncertainty of birth and the uniqueness of each birth, melding art, and science to plan and deliver the most appropriate care.

A story of confidence and courage, and its link to spirituality:

My grandmother used to say that God only gives you challenges you can take. That woman came in the clinics with substance-abuse issues and the others lamented: “Oh no, she comes every week!” But I went, I took the time, I brought her tea, then found something else to go see her. At one point she was in a lot of pain, so I offered her a bath. “I can have a bath?” It was in there that she started crying and opened up about her worries about the fact that she was a bad mother, etc.. I told her, “There is nothing we can do about the past, but you could things changed now.” [emotional] It takes courage to not stay with the colleagues at the desk, laughing and talking, but sometimes you have to go and make a difference in someone's life. Abby

Courage and confidence were not noted by the spiritual care providers encountered by Cherblanc and Jobin. And yet, it takes confidence to practice the profession of spiritual provider in a social context where spirituality is taboo, like it is in the Quebec context of their study, when one's credibility is constantly questioned and evaluated by other professionals, even by patients; it takes courage to defend a non-interventionist position, use intuition and trust the abilities of the person being supported. This might explain why in Cherblanc and Jobin's study, the most important indicator of success in practice stories is that of observation of a positive change in the person (for example, the person is calmer, more peaceful), then the positive recognition expressed by patients, families, or other health professionals. Paradoxically, even if Cherblanc and Jobin's paper do not address how spiritual care providers can continuously support other people in their suffering, spirituality is for many of them what fuels their confidence and courage.

### Human Relationship

Globally, the “human relationship” as the “glue” of the art of midwifery of MacLellan weaving all four of the other concepts as a prerequisite. A reciprocal human relationship of quality is based on compassion, openness, and kindness in communication; it conveys quietness and calmness. With this connection, empowerment of the woman is possible where she feels supported and in control.

This story told by an experienced midwife illustrates the centrality of human relationships in enabling the four other skills (presence, guardianship, intuition, and courage and confidence).

A few years ago, I helped this woman.

As I came in, there was a medical student, quite excitable: “Come on– push.” But there wasn’t any need for a rush; we didn’t need to be making this situation seems like there was a technological side of an urgency of doing – and in that doing, there was a busyness to do something, and the busyness and the doing were taking over the fact this woman was having a baby, it had taken stock of what her space, aura, feelings represented.

What that situation demanded and warranted, and needed to be propelled, was the woman. I did a few things; talking to the woman, giving her some space, finding out what was needed. I turned the lights down, and calmed the room down, and explained that everything was fine. I facilitated that connection of the sister with the mother in this space of having a baby; that was really what the woman wanted; I could sense this pressure with the sister, I said to her: “Shut your eyes, you will see everything you need to see, just zone in – listen to your sister – feel what's happening – you can feel baby's head under your hands – just concentrate – it will all be fine.”

We held time. I can just shut my eyes and be back there. I can remember the feelings. I can remember the change in the room when I turned off these bright lights and spotlighting everything back to the woman, within a soft haze of emotion and connection that was very, very different.

That was the best moment ever; it will live with me till I die, because that really cemented in me the power that I have, that I can really hold space: “Wow, I’ve brought this experience back for the woman.” There was a real intense connection, I didn’t require a lot of explanation: I zoned in, I connected, and I could feel what was happening because I was present and there were subtle clues of the way that she looked, the gestures of the woman's face. I was in the here and now – in the presence.

I was there for the woman; it was so beautiful that I’d helped her. It can be challenging to walk into those situations where time is of the essence: you can upset the dynamics in that room, but sometimes it can be the best thing for that woman and baby. I don’t mean it to sound like I’m cocky or bragging. We are gifted to be in this presence, and in that, we should be supporting that presence. Cathie

This characteristic originates from a philosophical stance or belief in the ability of women to birth their babies and reveals the centrality of the human relationship. Similarly, the notion of empowerment of the person is the key characteristic of the spiritual practice described by the spiritual care workers met by Cherblanc and Jobin, that is the establishment of a bond of trust with the person, to support them into connecting to their bio-psycho-social and in particular spiritual resources, all with the aim of allowing them to face as peacefully as possible the suffering and losses caused by illness and dead. Here, spirituality is represented as a resource that a person can mobilize. Like the concept of human relationship “glues” the four other characteristics of the art of midwifery practice, the notion of empowerment emerged during the analysis of spiritual intervention practices, through the numerous aims described but ultimately quite similar in substance: they are all marked by the idea to allow the person to find within themselves the resources allowing them to adapt to change and feel better.

Empowerment is a notion that is very common in health and social services but spiritual care providers in the Quebec healthcare system consider that no other care provider has the time, and the knowledge to do so; nor hold this shared egalitarian position. Behind this is revealed a deliberately resistance discourse against utilitarianism and the standardization of current hospital practices. Thus, in a hyper-segmented and hyper-competitive hospital paradigm between professions, each of them having to prove its problem-oriented effectiveness, the spiritual care providers hold onto their original non-directive position, foreign to a “productivist” perspective to assert their professional specificity. This brings us back to the aim shared by MacLellan of valorization of soft skills of the “art of midwifery practice” theoretical model; that sees the person as a whole, no longer the one receiving care, passively, but rather guiding his/her own process in the presence of the care giver. It is a call for professional humility yet resistance, as Hanna expresses, by giving the example of Shifra and Puah from the Hebrew Scriptures (Exodus 1) which describes the midwife as ‘god-fearing’ and morally exemplary in disobeying the King's command to kill the children:

She was wanting me to pray for her but I couldn’t take that spot in her: it would have just elevated me to a spot that God should have been in. Exodus 1 talks about when the midwives walked in the fear of God, and they valued all life, God was good to that nation and He made it mighty. Let's provide amazing emotional and physical care, but come back to the fear of God, for much better birthing outcomes. Hanna

Here fear is not being afraid, but moral conviction. Shifra and Puah exercise agency, moral courage providing an early example of civil disobedience within patriarchal and unjust systems of authority (believed to be first written between the 10th and 5th centuries BCE). The fear of God could be understood in this context as divine justice over organisational and human made authority where what matters most is foregrounded, revealing the sanctity and preciousness of life through human relationships.

## Discussion

The method used here reveals how this “art of midwifery practice” covers some important and already well-discussed characteristics of spiritual care, such as human relationship and presence, but also offers a new perspective on other characteristic only alluded in the “practiced spirituality” described by spiritual care providers in Cherblanc and Jobin's study. For spiritual care providers, this means that a deepened sense of some central aspect of the profession could be explored. For example, intuition is barely discussed and studied in spiritual care in healthcare contexts (Pesut et al., 2012) but daily used, and guardianship is not a concept developed in the profession. More globally, this characteristic is seen in the spiritual care literature, to different extent, but could benefit by the inspiration provided by midwifery.

For midwives, although the “art of midwifery practice” covers a lot of what spiritual looks like, despite research on spirituality around childbirth and national regulations stipulating the spiritual dimension of the profession, it has yet to be implemented in the midwifery educational curriculum. (Crowther et al., 2021; Hall, 2016; Hall & Crowther, 2021) Midwives are not talking about spirituality overtly, there is a tension round faith (Linhares, 2012); whereas it is the core of spiritual care practice: hence the latter could support midwifery by being more present in the educational process. This is also true in practice, where interprofessional collaboration is all too often limited to a call made by midwives to the palliative care chaplaincy/spiritual care service when death occurs around birth; this collaboration could be extended.

Through the crafted stories, the lived experiences of spiritual care of the midwives of our study offered a distinct approach for professional comparison, where other midwives but also spiritual care providers might recognize a description of their daily work or similar spiritual experiences in their practice. For both the midwife and the spiritual care worker, when supporting people throughout important life events, the concern is to create a connection to others and to allow reciprocity, trust, equality and understanding. Globally, midwives and spiritual care workers share the similar challenge of remaining at the interface between the health system (at the organizational/structural level) and the belief system (at the individual level); both professions navigate, with varying confidence and courage, to preserve (“be a guardian”) of presence and their intuition is to center the human relationship. To safeguard something is to ensure it is able to continue (Heidegger, 1927/1962); the ‘something’, alluded to is the innate special spiritual quality of mood in and around childbirth that consciously and unconsciously attune to a comportment in midwifery practice that shelters or protects that quality. (Crowther et al., 2014) For spiritual care providers this safeguarding is concerned with sheltering that which is significant in human relationships and perhaps includes connection with ineffable otherness. For the two professions, a tension remains with the dominant discourse, focused mainly on safety and pain management in the world of birth but more broadly located in a certain technoscientific and bureaucratic logic of the health establishment (Cherblanc & Jobin, 2020). This involves both developing an action (technical skill) recognized as “credible” by the health institution (with its own standards) while preserving the quality of being in the relationship, at the heart of the support that led to professionalization, the necessary soft skills described in MacLellan's theoretical model.

### Limitations

This study's limitations include the limits of the chosen theoretical models, notably as they did not share the same methodological creation process, midwifery's one being a discourse analysis of literature whereas the spiritual care's theoretical model was supported by a qualitative data collection. Moreover, the latter was geographically limited to Quebec spiritual care providers’ self-definition which might not be the same as elsewhere but also to those working in healthcare settings which might differ to jail or military chaplaincy. Also, Quebec and Aotearoa/New Zealand have similar social context in terms of spirituality which differs to other cultures: both are secular regions, largely characterized by the demise of religion with societies challenged to better include its indigenous population and increasingly cultural diversity. Finally, while providing some illustrative crafted stories around spiritual care within Aotearoa New Zealand midwifery, we are cognisant that this data was collected for a hermeneutic phenomenological study on presence as spiritual care and not explicitly a comparative focused study. As such a dedicated comparative study design that focuses on both midwifery and spiritual care providers is warranted. However, the secondary analysis of the crafted stories in this article has provided insightful comparations that contribute to our understanding of this domain.

## Conclusion

This paper is one of the first to compare spiritual care to midwifery's characteristics. By comparing theoretical models, it was observed that the characteristics of the art of midwifery practice could be perceived in the “practiced spirituality” of the spiritual care model. As such, spiritual care in healthcare can open its horizon of understanding by comparing its practice to professions outside the limited space of “psychosocial services”. In this article midwifery has shown that it can open the possibility to explore less discussed aspects of spiritual care provider's praxis by incorporating purposeful reflection on intuition, guardianship and courage.

### Implications

This study shows implications for practice, education, and research, as midwifery and spiritual care learn from one another. At this development stage as a healthcare profession, spiritual care can broaden its professional vision from entering into dialogue with other professions around common concerns (empowerment, presence, etc.) on which they sometimes have deeper or different perspectives and on which more research has been conducted. To deepen its educational and clinical practice, midwives could turn to spiritual care and include already-conducted studies on childbirth's spirituality. Further research could measure the impact of shared spaces for midwives and spiritual care providers such as conferences or shared discussions on complex clinical situations. It is evident that proactively working towards better inter-professional communications, as the authors of this article have accomplished, enhances understanding and appreciation of own profession.
